# Supplementary material for: Pyruvate Dehydrogenase Kinase 1 inhibition mediated oxidative phosphorylation enhancement in cartilage promotes osteoarthritis progression
Source: BMC Musculoskelet Disord. 2023 Jul 20;24:597. doi: 10.1186/s12891-023-06585-6 (PMC10357736; doi:10.1186/s12891-023-06585-6)
Supplement: Supplementary file 1 — Additional file 1. [file 12891_2023_6585_MOESM1_ESM.zip › BP.Gsea.1653623667859/GOBP_REPRODUCTIVE_SYSTEM_DEVELOPMENT.html]

Details for gene set GOBP\_REPRODUCTIVE\_SYSTEM\_DEVELOPMENT[GSEA]

|  || Dataset | 55457\_collapsed\_to\_symbols.cls.cls #OA\_versus\_normal.cls.cls #OA\_versus\_normal\_repos |
| Phenotype | cls.cls#OA\_versus\_normal\_repos |
| Upregulated in class | normal |
| GeneSet | GOBP\_REPRODUCTIVE\_SYSTEM\_DEVELOPMENT |
| Enrichment Score (ES) | -0.3360816 |
| Normalized Enrichment Score (NES) | -1.7374842 |
| Nominal p-value | 0.0 |
| FDR q-value | 0.12042137 |
| FWER p-Value | 1.0 |
Table: GSEA Results Summary

  

Fig 1: Enrichment plot: GOBP\_REPRODUCTIVE\_SYSTEM\_DEVELOPMENT      
 Profile of the Running ES Score & Positions of GeneSet Members on the Rank Ordered List

  

| SYMBOL | TITLE | RANK IN GENE LIST | RANK METRIC SCORE | RUNNING ES | CORE ENRICHMENT || 1 | PRDX4 | peroxiredoxin 4 [Source:HGNC Symbol;Acc:HGNC:17169] | 68 | 0.959 | 0.0030 | No |
| 2 | RTCB | "RNA 2',3'-cyclic phosphate and 5'-OH ligase [Source:HGNC Symbol;Acc:HGNC:26935]" | 78 | 0.947 | 0.0107 | No |
| 3 | TLR3 | toll like receptor 3 [Source:HGNC Symbol;Acc:HGNC:11849] | 175 | 0.837 | 0.0104 | No |
| 4 | PTN | pleiotrophin [Source:HGNC Symbol;Acc:HGNC:9630] | 402 | 0.706 | -0.0014 | No |
| 5 | SPINT2 | "serine peptidase inhibitor, Kunitz type 2 [Source:HGNC Symbol;Acc:HGNC:11247]" | 541 | 0.645 | -0.0068 | No |
| 6 | GJB3 | gap junction protein beta 3 [Source:HGNC Symbol;Acc:HGNC:4285] | 549 | 0.641 | -0.0017 | No |
| 7 | EIF2B2 | eukaryotic translation initiation factor 2B subunit beta [Source:HGNC Symbol;Acc:HGNC:3258] | 627 | 0.612 | -0.0024 | No |
| 8 | HESX1 | HESX homeobox 1 [Source:HGNC Symbol;Acc:HGNC:4877] | 656 | 0.604 | 0.0007 | No |
| 9 | IRX5 | iroquois homeobox 5 [Source:HGNC Symbol;Acc:HGNC:14361] | 733 | 0.585 | -0.0003 | No |
| 10 | MSH2 | mutS homolog 2 [Source:HGNC Symbol;Acc:HGNC:7325] | 739 | 0.584 | 0.0045 | No |
| 11 | GDF7 | growth differentiation factor 7 [Source:HGNC Symbol;Acc:HGNC:4222] | 928 | 0.533 | -0.0058 | No |
| 12 | ESR1 | estrogen receptor 1 [Source:HGNC Symbol;Acc:HGNC:3467] | 984 | 0.522 | -0.0056 | No |
| 13 | LDOC1 | LDOC1 regulator of NFKB signaling [Source:HGNC Symbol;Acc:HGNC:6548] | 989 | 0.520 | -0.0013 | No |
| 14 | AKT1 | AKT serine/threonine kinase 1 [Source:HGNC Symbol;Acc:HGNC:391] | 1192 | 0.478 | -0.0133 | No |
| 15 | HSD17B4 | hydroxysteroid 17-beta dehydrogenase 4 [Source:HGNC Symbol;Acc:HGNC:5213] | 1354 | 0.450 | -0.0221 | No |
| 16 | NUDT1 | nudix hydrolase 1 [Source:HGNC Symbol;Acc:HGNC:8048] | 1425 | 0.439 | -0.0239 | No |
| 17 | UBE2Q1 | ubiquitin conjugating enzyme E2 Q1 [Source:HGNC Symbol;Acc:HGNC:15698] | 1651 | 0.406 | -0.0382 | No |
| 18 | CEBPA | CCAAT enhancer binding protein alpha [Source:HGNC Symbol;Acc:HGNC:1833] | 1750 | 0.392 | -0.0426 | No |
| 19 | HOXA11 | homeobox A11 [Source:HGNC Symbol;Acc:HGNC:5101] | 1755 | 0.390 | -0.0395 | No |
| 20 | MEA1 | male-enhanced antigen 1 [Source:HGNC Symbol;Acc:HGNC:6986] | 1807 | 0.384 | -0.0402 | No |
| 21 | FGF9 | fibroblast growth factor 9 [Source:HGNC Symbol;Acc:HGNC:3687] | 1833 | 0.380 | -0.0388 | No |
| 22 | EAF2 | ELL associated factor 2 [Source:HGNC Symbol;Acc:HGNC:23115] | 1922 | 0.370 | -0.0426 | No |
| 23 | SRD5A1 | steroid 5 alpha-reductase 1 [Source:HGNC Symbol;Acc:HGNC:11284] | 1973 | 0.364 | -0.0434 | No |
| 24 | WNT5A | Wnt family member 5A [Source:HGNC Symbol;Acc:HGNC:12784] | 1995 | 0.361 | -0.0419 | No |
| 25 | AKR1C3 | aldo-keto reductase family 1 member C3 [Source:HGNC Symbol;Acc:HGNC:386] | 1996 | 0.361 | -0.0387 | No |
| 26 | GFRA1 | GDNF family receptor alpha 1 [Source:HGNC Symbol;Acc:HGNC:4243] | 2005 | 0.360 | -0.0361 | No |
| 27 | ANG | angiogenin [Source:HGNC Symbol;Acc:HGNC:483] | 2009 | 0.360 | -0.0332 | No |
| 28 | TPPP3 | tubulin polymerization promoting protein family member 3 [Source:HGNC Symbol;Acc:HGNC:24162] | 2041 | 0.357 | -0.0325 | No |
| 29 | SULF1 | sulfatase 1 [Source:HGNC Symbol;Acc:HGNC:20391] | 2071 | 0.354 | -0.0317 | No |
| 30 | SOX9 | SRY-box transcription factor 9 [Source:HGNC Symbol;Acc:HGNC:11204] | 2089 | 0.351 | -0.0300 | No |
| 31 | GRHL2 | grainyhead like transcription factor 2 [Source:HGNC Symbol;Acc:HGNC:2799] | 2111 | 0.348 | -0.0286 | No |
| 32 | KIT | "KIT proto-oncogene, receptor tyrosine kinase [Source:HGNC Symbol;Acc:HGNC:6342]" | 2117 | 0.348 | -0.0259 | No |
| 33 | PRDX3 | peroxiredoxin 3 [Source:HGNC Symbol;Acc:HGNC:9354] | 2161 | 0.343 | -0.0263 | No |
| 34 | SRY | sex determining region Y [Source:HGNC Symbol;Acc:HGNC:11311] | 2211 | 0.337 | -0.0273 | No |
| 35 | KRT19 | keratin 19 [Source:HGNC Symbol;Acc:HGNC:6436] | 2258 | 0.331 | -0.0280 | No |
| 36 | CRKL | "CRK like proto-oncogene, adaptor protein [Source:HGNC Symbol;Acc:HGNC:2363]" | 2283 | 0.329 | -0.0270 | No |
| 37 | MAPK14 | mitogen-activated protein kinase 14 [Source:HGNC Symbol;Acc:HGNC:6876] | 2352 | 0.320 | -0.0296 | No |
| 38 | HOXA10 | homeobox A10 [Source:HGNC Symbol;Acc:HGNC:5100] | 2408 | 0.313 | -0.0313 | No |
| 39 | SOD1 | superoxide dismutase 1 [Source:HGNC Symbol;Acc:HGNC:11179] | 2413 | 0.313 | -0.0288 | No |
| 40 | ROR2 | receptor tyrosine kinase like orphan receptor 2 [Source:HGNC Symbol;Acc:HGNC:10257] | 2448 | 0.308 | -0.0288 | No |
| 41 | SFRP1 | secreted frizzled related protein 1 [Source:HGNC Symbol;Acc:HGNC:10776] | 2503 | 0.303 | -0.0305 | No |
| 42 | ACSBG1 | acyl-CoA synthetase bubblegum family member 1 [Source:HGNC Symbol;Acc:HGNC:29567] | 2520 | 0.301 | -0.0291 | No |
| 43 | GREB1L | GREB1 like retinoic acid receptor coactivator [Source:HGNC Symbol;Acc:HGNC:31042] | 2524 | 0.300 | -0.0267 | No |
| 44 | WNT2 | Wnt family member 2 [Source:HGNC Symbol;Acc:HGNC:12780] | 2700 | 0.279 | -0.0382 | No |
| 45 | SOX15 | SRY-box transcription factor 15 [Source:HGNC Symbol;Acc:HGNC:11196] | 2748 | 0.275 | -0.0395 | No |
| 46 | C3 | complement C3 [Source:HGNC Symbol;Acc:HGNC:1318] | 2764 | 0.273 | -0.0383 | No |
| 47 | GLI3 | GLI family zinc finger 3 [Source:HGNC Symbol;Acc:HGNC:4319] | 2861 | 0.264 | -0.0436 | No |
| 48 | GAS2 | growth arrest specific 2 [Source:HGNC Symbol;Acc:HGNC:4167] | 2866 | 0.264 | -0.0416 | No |
| 49 | PDGFRA | platelet derived growth factor receptor alpha [Source:HGNC Symbol;Acc:HGNC:8803] | 2943 | 0.256 | -0.0454 | No |
| 50 | ERCC1 | "ERCC excision repair 1, endonuclease non-catalytic subunit [Source:HGNC Symbol;Acc:HGNC:3433]" | 2948 | 0.255 | -0.0435 | No |
| 51 | CDKN1B | cyclin dependent kinase inhibitor 1B [Source:HGNC Symbol;Acc:HGNC:1785] | 2984 | 0.251 | -0.0441 | No |
| 52 | GATA4 | GATA binding protein 4 [Source:HGNC Symbol;Acc:HGNC:4173] | 3009 | 0.249 | -0.0438 | No |
| 53 | NTRK1 | neurotrophic receptor tyrosine kinase 1 [Source:HGNC Symbol;Acc:HGNC:8031] | 3015 | 0.248 | -0.0420 | No |
| 54 | SENP2 | SUMO specific peptidase 2 [Source:HGNC Symbol;Acc:HGNC:23116] | 3040 | 0.246 | -0.0418 | No |
| 55 | BIK | BCL2 interacting killer [Source:HGNC Symbol;Acc:HGNC:1051] | 3057 | 0.244 | -0.0409 | No |
| 56 | PCDH12 | protocadherin 12 [Source:HGNC Symbol;Acc:HGNC:8657] | 3081 | 0.242 | -0.0406 | No |
| 57 | PLAG1 | PLAG1 zinc finger [Source:HGNC Symbol;Acc:HGNC:9045] | 3095 | 0.240 | -0.0395 | No |
| 58 | SEMA3A | semaphorin 3A [Source:HGNC Symbol;Acc:HGNC:10723] | 3104 | 0.239 | -0.0380 | No |
| 59 | DHCR24 | 24-dehydrocholesterol reductase [Source:HGNC Symbol;Acc:HGNC:2859] | 3126 | 0.237 | -0.0376 | No |
| 60 | TFEB | transcription factor EB [Source:HGNC Symbol;Acc:HGNC:11753] | 3190 | 0.232 | -0.0406 | No |
| 61 | CASP3 | caspase 3 [Source:HGNC Symbol;Acc:HGNC:1504] | 3202 | 0.230 | -0.0395 | No |
| 62 | TFPT | TCF3 fusion partner [Source:HGNC Symbol;Acc:HGNC:13630] | 3226 | 0.228 | -0.0393 | No |
| 63 | LRP2 | LDL receptor related protein 2 [Source:HGNC Symbol;Acc:HGNC:6694] | 3236 | 0.227 | -0.0380 | No |
| 64 | FRS2 | fibroblast growth factor receptor substrate 2 [Source:HGNC Symbol;Acc:HGNC:16971] | 3385 | 0.213 | -0.0480 | No |
| 65 | PTPN6 | protein tyrosine phosphatase non-receptor type 6 [Source:HGNC Symbol;Acc:HGNC:9658] | 3434 | 0.208 | -0.0500 | No |
| 66 | CRIP1 | cysteine rich protein 1 [Source:HGNC Symbol;Acc:HGNC:2360] | 3446 | 0.207 | -0.0490 | No |
| 67 | SGPL1 | sphingosine-1-phosphate lyase 1 [Source:HGNC Symbol;Acc:HGNC:10817] | 3462 | 0.206 | -0.0484 | No |
| 68 | TRIM28 | tripartite motif containing 28 [Source:HGNC Symbol;Acc:HGNC:16384] | 3526 | 0.200 | -0.0517 | No |
| 69 | VDR | vitamin D receptor [Source:HGNC Symbol;Acc:HGNC:12679] | 3571 | 0.197 | -0.0534 | No |
| 70 | CYP7B1 | cytochrome P450 family 7 subfamily B member 1 [Source:HGNC Symbol;Acc:HGNC:2652] | 3584 | 0.195 | -0.0527 | No |
| 71 | MAS1 | "MAS1 proto-oncogene, G protein-coupled receptor [Source:HGNC Symbol;Acc:HGNC:6899]" | 3629 | 0.191 | -0.0545 | No |
| 72 | CSF2 | colony stimulating factor 2 [Source:HGNC Symbol;Acc:HGNC:2434] | 3699 | 0.185 | -0.0584 | No |
| 73 | BRCA2 | BRCA2 DNA repair associated [Source:HGNC Symbol;Acc:HGNC:1101] | 3853 | 0.170 | -0.0691 | No |
| 74 | EIF2B4 | eukaryotic translation initiation factor 2B subunit delta [Source:HGNC Symbol;Acc:HGNC:3260] | 3878 | 0.167 | -0.0695 | No |
| 75 | ST14 | ST14 transmembrane serine protease matriptase [Source:HGNC Symbol;Acc:HGNC:11344] | 3900 | 0.165 | -0.0698 | No |
| 76 | SALL1 | spalt like transcription factor 1 [Source:HGNC Symbol;Acc:HGNC:10524] | 3930 | 0.162 | -0.0706 | No |
| 77 | SPINT1 | "serine peptidase inhibitor, Kunitz type 1 [Source:HGNC Symbol;Acc:HGNC:11246]" | 3938 | 0.161 | -0.0698 | No |
| 78 | ZFPM2 | "zinc finger protein, FOG family member 2 [Source:HGNC Symbol;Acc:HGNC:16700]" | 3958 | 0.160 | -0.0699 | No |
| 79 | CTNNA1 | catenin alpha 1 [Source:HGNC Symbol;Acc:HGNC:2509] | 4003 | 0.156 | -0.0720 | No |
| 80 | HEY2 | hes related family bHLH transcription factor with YRPW motif 2 [Source:HGNC Symbol;Acc:HGNC:4881] | 4058 | 0.150 | -0.0750 | No |
| 81 | DMC1 | DNA meiotic recombinase 1 [Source:HGNC Symbol;Acc:HGNC:2927] | 4149 | 0.142 | -0.0809 | No |
| 82 | MKKS | MKKS centrosomal shuttling protein [Source:HGNC Symbol;Acc:HGNC:7108] | 4182 | 0.140 | -0.0823 | No |
| 83 | WNT2B | Wnt family member 2B [Source:HGNC Symbol;Acc:HGNC:12781] | 4194 | 0.138 | -0.0819 | No |
| 84 | SPP1 | secreted phosphoprotein 1 [Source:HGNC Symbol;Acc:HGNC:11255] | 4205 | 0.137 | -0.0815 | No |
| 85 | CDKN1C | cyclin dependent kinase inhibitor 1C [Source:HGNC Symbol;Acc:HGNC:1786] | 4233 | 0.134 | -0.0825 | No |
| 86 | GDF9 | growth differentiation factor 9 [Source:HGNC Symbol;Acc:HGNC:4224] | 4318 | 0.127 | -0.0881 | No |
| 87 | TCF21 | transcription factor 21 [Source:HGNC Symbol;Acc:HGNC:11632] | 4321 | 0.127 | -0.0871 | No |
| 88 | PRLR | prolactin receptor [Source:HGNC Symbol;Acc:HGNC:9446] | 4323 | 0.126 | -0.0861 | No |
| 89 | VASH2 | vasohibin 2 [Source:HGNC Symbol;Acc:HGNC:25723] | 4345 | 0.124 | -0.0867 | No |
| 90 | AGO4 | argonaute RISC component 4 [Source:HGNC Symbol;Acc:HGNC:18424] | 4346 | 0.124 | -0.0856 | No |
| 91 | ZP3 | zona pellucida glycoprotein 3 [Source:HGNC Symbol;Acc:HGNC:13189] | 4367 | 0.123 | -0.0861 | No |
| 92 | IGF2 | insulin like growth factor 2 [Source:HGNC Symbol;Acc:HGNC:5466] | 4378 | 0.123 | -0.0858 | No |
| 93 | FSHB | follicle stimulating hormone subunit beta [Source:HGNC Symbol;Acc:HGNC:3964] | 4502 | 0.113 | -0.0946 | No |
| 94 | EPOR | erythropoietin receptor [Source:HGNC Symbol;Acc:HGNC:3416] | 4596 | 0.105 | -0.1011 | No |
| 95 | WDR48 | WD repeat domain 48 [Source:HGNC Symbol;Acc:HGNC:30914] | 4735 | 0.092 | -0.1113 | No |
| 96 | FGF8 | fibroblast growth factor 8 [Source:HGNC Symbol;Acc:HGNC:3686] | 4761 | 0.090 | -0.1125 | No |
| 97 | ALKBH1 | "alkB homolog 1, histone H2A dioxygenase [Source:HGNC Symbol;Acc:HGNC:17911]" | 4765 | 0.090 | -0.1120 | No |
| 98 | TBX3 | T-box transcription factor 3 [Source:HGNC Symbol;Acc:HGNC:11602] | 4790 | 0.088 | -0.1131 | No |
| 99 | HOXD13 | homeobox D13 [Source:HGNC Symbol;Acc:HGNC:5136] | 4837 | 0.084 | -0.1160 | No |
| 100 | EIF2S2 | eukaryotic translation initiation factor 2 subunit beta [Source:HGNC Symbol;Acc:HGNC:3266] | 4838 | 0.084 | -0.1153 | No |
| 101 | SERPINE2 | serpin family E member 2 [Source:HGNC Symbol;Acc:HGNC:8951] | 4873 | 0.082 | -0.1173 | No |
| 102 | TNC | tenascin C [Source:HGNC Symbol;Acc:HGNC:5318] | 4874 | 0.082 | -0.1166 | No |
| 103 | BASP1 | brain abundant membrane attached signal protein 1 [Source:HGNC Symbol;Acc:HGNC:957] | 4923 | 0.078 | -0.1197 | No |
| 104 | OVOL2 | ovo like zinc finger 2 [Source:HGNC Symbol;Acc:HGNC:15804] | 5017 | 0.071 | -0.1265 | No |
| 105 | AMH | anti-Mullerian hormone [Source:HGNC Symbol;Acc:HGNC:464] | 5042 | 0.070 | -0.1278 | No |
| 106 | BIRC2 | baculoviral IAP repeat containing 2 [Source:HGNC Symbol;Acc:HGNC:590] | 5054 | 0.069 | -0.1281 | No |
| 107 | MSH4 | mutS homolog 4 [Source:HGNC Symbol;Acc:HGNC:7327] | 5055 | 0.069 | -0.1275 | No |
| 108 | RBP4 | retinol binding protein 4 [Source:HGNC Symbol;Acc:HGNC:9922] | 5072 | 0.067 | -0.1282 | No |
| 109 | MERTK | "MER proto-oncogene, tyrosine kinase [Source:HGNC Symbol;Acc:HGNC:7027]" | 5085 | 0.066 | -0.1285 | No |
| 110 | SLIT3 | slit guidance ligand 3 [Source:HGNC Symbol;Acc:HGNC:11087] | 5134 | 0.061 | -0.1318 | No |
| 111 | ASPM | assembly factor for spindle microtubules [Source:HGNC Symbol;Acc:HGNC:19048] | 5164 | 0.059 | -0.1336 | No |
| 112 | ASCL2 | achaete-scute family bHLH transcription factor 2 [Source:HGNC Symbol;Acc:HGNC:739] | 5194 | 0.057 | -0.1355 | No |
| 113 | TSPY1 | testis specific protein Y-linked 1 [Source:HGNC Symbol;Acc:HGNC:12381] | 5214 | 0.055 | -0.1365 | No |
| 114 | FOXC1 | forkhead box C1 [Source:HGNC Symbol;Acc:HGNC:3800] | 5227 | 0.054 | -0.1370 | No |
| 115 | HMGB2 | high mobility group box 2 [Source:HGNC Symbol;Acc:HGNC:5000] | 5272 | 0.050 | -0.1400 | No |
| 116 | TGFBR1 | transforming growth factor beta receptor 1 [Source:HGNC Symbol;Acc:HGNC:11772] | 5280 | 0.050 | -0.1402 | No |
| 117 | FZD5 | frizzled class receptor 5 [Source:HGNC Symbol;Acc:HGNC:4043] | 5392 | 0.041 | -0.1487 | No |
| 118 | FNDC3A | fibronectin type III domain containing 3A [Source:HGNC Symbol;Acc:HGNC:20296] | 5403 | 0.040 | -0.1491 | No |
| 119 | WT1 | WT1 transcription factor [Source:HGNC Symbol;Acc:HGNC:12796] | 5452 | 0.037 | -0.1526 | No |
| 120 | KDM5B | lysine demethylase 5B [Source:HGNC Symbol;Acc:HGNC:18039] | 5484 | 0.033 | -0.1548 | No |
| 121 | SDC1 | syndecan 1 [Source:HGNC Symbol;Acc:HGNC:10658] | 5526 | 0.031 | -0.1578 | No |
| 122 | E2F8 | E2F transcription factor 8 [Source:HGNC Symbol;Acc:HGNC:24727] | 5527 | 0.031 | -0.1575 | No |
| 123 | GATA6 | GATA binding protein 6 [Source:HGNC Symbol;Acc:HGNC:4174] | 5537 | 0.030 | -0.1580 | No |
| 124 | GJB5 | gap junction protein beta 5 [Source:HGNC Symbol;Acc:HGNC:4287] | 5542 | 0.029 | -0.1580 | No |
| 125 | STAT5B | signal transducer and activator of transcription 5B [Source:HGNC Symbol;Acc:HGNC:11367] | 5554 | 0.028 | -0.1587 | No |
| 126 | BCL2 | BCL2 apoptosis regulator [Source:HGNC Symbol;Acc:HGNC:990] | 5609 | 0.023 | -0.1628 | No |
| 127 | NUPR1 | "nuclear protein 1, transcriptional regulator [Source:HGNC Symbol;Acc:HGNC:29990]" | 5616 | 0.023 | -0.1631 | No |
| 128 | GNRH1 | gonadotropin releasing hormone 1 [Source:HGNC Symbol;Acc:HGNC:4419] | 5621 | 0.022 | -0.1632 | No |
| 129 | BCL2L2 | BCL2 like 2 [Source:HGNC Symbol;Acc:HGNC:995] | 5643 | 0.020 | -0.1647 | No |
| 130 | FANCG | FA complementation group G [Source:HGNC Symbol;Acc:HGNC:3588] | 5675 | 0.017 | -0.1670 | No |
| 131 | LHCGR | luteinizing hormone/choriogonadotropin receptor [Source:HGNC Symbol;Acc:HGNC:6585] | 5679 | 0.017 | -0.1671 | No |
| 132 | GRB2 | growth factor receptor bound protein 2 [Source:HGNC Symbol;Acc:HGNC:4566] | 5697 | 0.016 | -0.1683 | No |
| 133 | INHA | inhibin subunit alpha [Source:HGNC Symbol;Acc:HGNC:6065] | 5701 | 0.016 | -0.1684 | No |
| 134 | TMED2 | transmembrane p24 trafficking protein 2 [Source:HGNC Symbol;Acc:HGNC:16996] | 5709 | 0.015 | -0.1688 | No |
| 135 | FSHR | follicle stimulating hormone receptor [Source:HGNC Symbol;Acc:HGNC:3969] | 5757 | 0.011 | -0.1725 | No |
| 136 | RNF38 | ring finger protein 38 [Source:HGNC Symbol;Acc:HGNC:18052] | 5774 | 0.010 | -0.1737 | No |
| 137 | PLEKHA5 | pleckstrin homology domain containing A5 [Source:HGNC Symbol;Acc:HGNC:30036] | 5865 | 0.004 | -0.1808 | No |
| 138 | BRIP1 | BRCA1 interacting helicase 1 [Source:HGNC Symbol;Acc:HGNC:20473] | 5885 | 0.003 | -0.1823 | No |
| 139 | ETNK2 | ethanolamine kinase 2 [Source:HGNC Symbol;Acc:HGNC:25575] | 5897 | 0.002 | -0.1832 | No |
| 140 | ZFY | zinc finger protein Y-linked [Source:HGNC Symbol;Acc:HGNC:12870] | 5909 | 0.002 | -0.1840 | No |
| 141 | SPRR2A | small proline rich protein 2A [Source:HGNC Symbol;Acc:HGNC:11261] | 5922 | 0.001 | -0.1850 | No |
| 142 | CYP1B1 | cytochrome P450 family 1 subfamily B member 1 [Source:HGNC Symbol;Acc:HGNC:2597] | 5925 | 0.000 | -0.1851 | No |
| 143 | BMPR1B | bone morphogenetic protein receptor type 1B [Source:HGNC Symbol;Acc:HGNC:1077] | 5959 | -0.002 | -0.1878 | No |
| 144 | ACVR2A | activin A receptor type 2A [Source:HGNC Symbol;Acc:HGNC:173] | 5960 | -0.002 | -0.1878 | No |
| 145 | PPARG | peroxisome proliferator activated receptor gamma [Source:HGNC Symbol;Acc:HGNC:9236] | 6006 | -0.005 | -0.1913 | No |
| 146 | ASB1 | ankyrin repeat and SOCS box containing 1 [Source:HGNC Symbol;Acc:HGNC:16011] | 6015 | -0.006 | -0.1919 | No |
| 147 | PLK4 | polo like kinase 4 [Source:HGNC Symbol;Acc:HGNC:11397] | 6041 | -0.008 | -0.1938 | No |
| 148 | FOXL2 | forkhead box L2 [Source:HGNC Symbol;Acc:HGNC:1092] | 6068 | -0.009 | -0.1958 | No |
| 149 | TYRO3 | TYRO3 protein tyrosine kinase [Source:HGNC Symbol;Acc:HGNC:12446] | 6102 | -0.013 | -0.1983 | No |
| 150 | ING2 | inhibitor of growth family member 2 [Source:HGNC Symbol;Acc:HGNC:6063] | 6150 | -0.016 | -0.2019 | No |
| 151 | LFNG | LFNG O-fucosylpeptide 3-beta-N-acetylglucosaminyltransferase [Source:HGNC Symbol;Acc:HGNC:6560] | 6161 | -0.017 | -0.2026 | No |
| 152 | LHX1 | LIM homeobox 1 [Source:HGNC Symbol;Acc:HGNC:6593] | 6164 | -0.017 | -0.2026 | No |
| 153 | ATM | ATM serine/threonine kinase [Source:HGNC Symbol;Acc:HGNC:795] | 6179 | -0.019 | -0.2036 | No |
| 154 | DLG1 | discs large MAGUK scaffold protein 1 [Source:HGNC Symbol;Acc:HGNC:2900] | 6219 | -0.022 | -0.2065 | No |
| 155 | WNT7B | Wnt family member 7B [Source:HGNC Symbol;Acc:HGNC:12787] | 6230 | -0.023 | -0.2071 | No |
| 156 | PKD2 | "polycystin 2, transient receptor potential cation channel [Source:HGNC Symbol;Acc:HGNC:9009]" | 6254 | -0.024 | -0.2087 | No |
| 157 | TP63 | tumor protein p63 [Source:HGNC Symbol;Acc:HGNC:15979] | 6274 | -0.025 | -0.2100 | No |
| 158 | GGNBP2 | gametogenetin binding protein 2 [Source:HGNC Symbol;Acc:HGNC:19357] | 6301 | -0.027 | -0.2118 | No |
| 159 | CYP19A1 | cytochrome P450 family 19 subfamily A member 1 [Source:HGNC Symbol;Acc:HGNC:2594] | 6388 | -0.034 | -0.2184 | No |
| 160 | KIF18A | kinesin family member 18A [Source:HGNC Symbol;Acc:HGNC:29441] | 6414 | -0.036 | -0.2201 | No |
| 161 | CCDC134 | coiled-coil domain containing 134 [Source:HGNC Symbol;Acc:HGNC:26185] | 6436 | -0.038 | -0.2214 | No |
| 162 | SERPINF1 | serpin family F member 1 [Source:HGNC Symbol;Acc:HGNC:8824] | 6492 | -0.042 | -0.2254 | No |
| 163 | LHX3 | LIM homeobox 3 [Source:HGNC Symbol;Acc:HGNC:6595] | 6495 | -0.042 | -0.2252 | No |
| 164 | INSL3 | insulin like 3 [Source:HGNC Symbol;Acc:HGNC:6086] | 6531 | -0.045 | -0.2276 | No |
| 165 | AFP | alpha fetoprotein [Source:HGNC Symbol;Acc:HGNC:317] | 6561 | -0.047 | -0.2295 | No |
| 166 | BCL2L1 | BCL2 like 1 [Source:HGNC Symbol;Acc:HGNC:992] | 6698 | -0.055 | -0.2399 | No |
| 167 | TTPA | alpha tocopherol transfer protein [Source:HGNC Symbol;Acc:HGNC:12404] | 6796 | -0.063 | -0.2471 | No |
| 168 | TNFAIP6 | TNF alpha induced protein 6 [Source:HGNC Symbol;Acc:HGNC:11898] | 6799 | -0.063 | -0.2467 | No |
| 169 | MAPK1 | mitogen-activated protein kinase 1 [Source:HGNC Symbol;Acc:HGNC:6871] | 6853 | -0.068 | -0.2503 | No |
| 170 | NR0B1 | nuclear receptor subfamily 0 group B member 1 [Source:HGNC Symbol;Acc:HGNC:7960] | 6885 | -0.070 | -0.2522 | No |
| 171 | PDGFRB | platelet derived growth factor receptor beta [Source:HGNC Symbol;Acc:HGNC:8804] | 6914 | -0.073 | -0.2538 | No |
| 172 | SETD2 | "SET domain containing 2, histone lysine methyltransferase [Source:HGNC Symbol;Acc:HGNC:18420]" | 6926 | -0.074 | -0.2540 | No |
| 173 | SRC | "SRC proto-oncogene, non-receptor tyrosine kinase [Source:HGNC Symbol;Acc:HGNC:11283]" | 6940 | -0.075 | -0.2544 | No |
| 174 | TGFB2 | transforming growth factor beta 2 [Source:HGNC Symbol;Acc:HGNC:11768] | 6985 | -0.079 | -0.2572 | No |
| 175 | ADAM21 | ADAM metallopeptidase domain 21 [Source:HGNC Symbol;Acc:HGNC:200] | 7010 | -0.081 | -0.2584 | No |
| 176 | SP3 | Sp3 transcription factor [Source:HGNC Symbol;Acc:HGNC:11208] | 7033 | -0.082 | -0.2594 | No |
| 177 | MMP2 | matrix metallopeptidase 2 [Source:HGNC Symbol;Acc:HGNC:7166] | 7037 | -0.082 | -0.2589 | No |
| 178 | SPATA2 | spermatogenesis associated 2 [Source:HGNC Symbol;Acc:HGNC:14681] | 7045 | -0.083 | -0.2588 | No |
| 179 | CITED2 | Cbp/p300 interacting transactivator with Glu/Asp rich carboxy-terminal domain 2 [Source:HGNC Symbol;Acc:HGNC:1987] | 7100 | -0.087 | -0.2623 | No |
| 180 | CUL7 | cullin 7 [Source:HGNC Symbol;Acc:HGNC:21024] | 7105 | -0.088 | -0.2618 | No |
| 181 | STRA6 | signaling receptor and transporter of retinol STRA6 [Source:HGNC Symbol;Acc:HGNC:30650] | 7107 | -0.089 | -0.2611 | No |
| 182 | IDH1 | isocitrate dehydrogenase (NADP(+)) 1 [Source:HGNC Symbol;Acc:HGNC:5382] | 7109 | -0.089 | -0.2604 | No |
| 183 | WNT7A | Wnt family member 7A [Source:HGNC Symbol;Acc:HGNC:12786] | 7166 | -0.093 | -0.2641 | No |
| 184 | ARRB2 | arrestin beta 2 [Source:HGNC Symbol;Acc:HGNC:712] | 7255 | -0.100 | -0.2702 | No |
| 185 | PTK2 | protein tyrosine kinase 2 [Source:HGNC Symbol;Acc:HGNC:9611] | 7315 | -0.103 | -0.2740 | No |
| 186 | H3-3B | H3.3 histone B [Source:HGNC Symbol;Acc:HGNC:4765] | 7351 | -0.106 | -0.2759 | No |
| 187 | ETV2 | ETS variant transcription factor 2 [Source:HGNC Symbol;Acc:HGNC:3491] | 7365 | -0.108 | -0.2760 | No |
| 188 | FGFR2 | fibroblast growth factor receptor 2 [Source:HGNC Symbol;Acc:HGNC:3689] | 7374 | -0.108 | -0.2757 | No |
| 189 | SPO11 | SPO11 initiator of meiotic double stranded breaks [Source:HGNC Symbol;Acc:HGNC:11250] | 7436 | -0.114 | -0.2795 | No |
| 190 | LEF1 | lymphoid enhancer binding factor 1 [Source:HGNC Symbol;Acc:HGNC:6551] | 7467 | -0.116 | -0.2809 | No |
| 191 | DMRT1 | doublesex and mab-3 related transcription factor 1 [Source:HGNC Symbol;Acc:HGNC:2934] | 7508 | -0.119 | -0.2831 | No |
| 192 | CCND1 | cyclin D1 [Source:HGNC Symbol;Acc:HGNC:1582] | 7512 | -0.119 | -0.2822 | No |
| 193 | CDX4 | caudal type homeobox 4 [Source:HGNC Symbol;Acc:HGNC:1808] | 7519 | -0.120 | -0.2817 | No |
| 194 | MEN1 | menin 1 [Source:HGNC Symbol;Acc:HGNC:7010] | 7542 | -0.122 | -0.2824 | No |
| 195 | ATRX | ATRX chromatin remodeler [Source:HGNC Symbol;Acc:HGNC:886] | 7551 | -0.123 | -0.2819 | No |
| 196 | ARID4A | AT-rich interaction domain 4A [Source:HGNC Symbol;Acc:HGNC:9885] | 7612 | -0.128 | -0.2856 | No |
| 197 | NHLH2 | nescient helix-loop-helix 2 [Source:HGNC Symbol;Acc:HGNC:7818] | 7626 | -0.129 | -0.2855 | No |
| 198 | WDR19 | WD repeat domain 19 [Source:HGNC Symbol;Acc:HGNC:18340] | 7627 | -0.129 | -0.2843 | No |
| 199 | NDP | norrin cystine knot growth factor NDP [Source:HGNC Symbol;Acc:HGNC:7678] | 7631 | -0.129 | -0.2834 | No |
| 200 | BMP4 | bone morphogenetic protein 4 [Source:HGNC Symbol;Acc:HGNC:1071] | 7816 | -0.142 | -0.2969 | No |
| 201 | IL1A | interleukin 1 alpha [Source:HGNC Symbol;Acc:HGNC:5991] | 7842 | -0.144 | -0.2976 | No |
| 202 | BMP15 | bone morphogenetic protein 15 [Source:HGNC Symbol;Acc:HGNC:1068] | 7889 | -0.147 | -0.3000 | No |
| 203 | MAMLD1 | mastermind like domain containing 1 [Source:HGNC Symbol;Acc:HGNC:2568] | 7949 | -0.151 | -0.3034 | No |
| 204 | TAF4 | TATA-box binding protein associated factor 4 [Source:HGNC Symbol;Acc:HGNC:11537] | 7966 | -0.153 | -0.3033 | No |
| 205 | FEM1B | fem-1 homolog B [Source:HGNC Symbol;Acc:HGNC:3649] | 8065 | -0.160 | -0.3097 | No |
| 206 | ARID4B | AT-rich interaction domain 4B [Source:HGNC Symbol;Acc:HGNC:15550] | 8078 | -0.161 | -0.3092 | No |
| 207 | ADA | adenosine deaminase [Source:HGNC Symbol;Acc:HGNC:186] | 8105 | -0.163 | -0.3099 | No |
| 208 | FZD4 | frizzled class receptor 4 [Source:HGNC Symbol;Acc:HGNC:4042] | 8159 | -0.168 | -0.3126 | No |
| 209 | EIF2B5 | eukaryotic translation initiation factor 2B subunit epsilon [Source:HGNC Symbol;Acc:HGNC:3261] | 8168 | -0.168 | -0.3118 | No |
| 210 | ID4 | "inhibitor of DNA binding 4, HLH protein [Source:HGNC Symbol;Acc:HGNC:5363]" | 8174 | -0.169 | -0.3107 | No |
| 211 | HEY1 | hes related family bHLH transcription factor with YRPW motif 1 [Source:HGNC Symbol;Acc:HGNC:4880] | 8213 | -0.172 | -0.3122 | No |
| 212 | AR | androgen receptor [Source:HGNC Symbol;Acc:HGNC:644] | 8217 | -0.173 | -0.3109 | No |
| 213 | PRPS1L1 | phosphoribosyl pyrophosphate synthetase 1 like 1 [Source:HGNC Symbol;Acc:HGNC:9463] | 8382 | -0.184 | -0.3224 | No |
| 214 | SLIT2 | slit guidance ligand 2 [Source:HGNC Symbol;Acc:HGNC:11086] | 8449 | -0.190 | -0.3260 | No |
| 215 | ADAM29 | ADAM metallopeptidase domain 29 [Source:HGNC Symbol;Acc:HGNC:207] | 8462 | -0.191 | -0.3253 | No |
| 216 | GHSR | growth hormone secretagogue receptor [Source:HGNC Symbol;Acc:HGNC:4267] | 8472 | -0.191 | -0.3243 | No |
| 217 | VASH1 | vasohibin 1 [Source:HGNC Symbol;Acc:HGNC:19964] | 8473 | -0.191 | -0.3226 | No |
| 218 | BMP5 | bone morphogenetic protein 5 [Source:HGNC Symbol;Acc:HGNC:1072] | 8488 | -0.193 | -0.3220 | No |
| 219 | FST | follistatin [Source:HGNC Symbol;Acc:HGNC:3971] | 8511 | -0.194 | -0.3221 | No |
| 220 | NRIP1 | nuclear receptor interacting protein 1 [Source:HGNC Symbol;Acc:HGNC:8001] | 8524 | -0.195 | -0.3213 | No |
| 221 | AXL | AXL receptor tyrosine kinase [Source:HGNC Symbol;Acc:HGNC:905] | 8565 | -0.199 | -0.3227 | No |
| 222 | PATZ1 | POZ/BTB and AT hook containing zinc finger 1 [Source:HGNC Symbol;Acc:HGNC:13071] | 8589 | -0.200 | -0.3228 | No |
| 223 | MAP3K4 | mitogen-activated protein kinase kinase kinase 4 [Source:HGNC Symbol;Acc:HGNC:6856] | 8637 | -0.203 | -0.3248 | No |
| 224 | RARG | retinoic acid receptor gamma [Source:HGNC Symbol;Acc:HGNC:9866] | 8700 | -0.209 | -0.3279 | No |
| 225 | LEP | leptin [Source:HGNC Symbol;Acc:HGNC:6553] | 8740 | -0.212 | -0.3291 | No |
| 226 | PKD1 | "polycystin 1, transient receptor potential channel interacting [Source:HGNC Symbol;Acc:HGNC:9008]" | 8755 | -0.213 | -0.3284 | No |
| 227 | UBE3A | ubiquitin protein ligase E3A [Source:HGNC Symbol;Acc:HGNC:12496] | 8757 | -0.213 | -0.3266 | No |
| 228 | RBPJ | recombination signal binding protein for immunoglobulin kappa J region [Source:HGNC Symbol;Acc:HGNC:5724] | 8800 | -0.217 | -0.3280 | No |
| 229 | TFAP2C | transcription factor AP-2 gamma [Source:HGNC Symbol;Acc:HGNC:11744] | 8824 | -0.219 | -0.3279 | No |
| 230 | PTCH1 | patched 1 [Source:HGNC Symbol;Acc:HGNC:9585] | 8837 | -0.220 | -0.3269 | No |
| 231 | IL10 | interleukin 10 [Source:HGNC Symbol;Acc:HGNC:5962] | 8855 | -0.221 | -0.3263 | No |
| 232 | A2M | alpha-2-macroglobulin [Source:HGNC Symbol;Acc:HGNC:7] | 8876 | -0.223 | -0.3260 | No |
| 233 | DEDD | death effector domain containing [Source:HGNC Symbol;Acc:HGNC:2755] | 8888 | -0.224 | -0.3249 | No |
| 234 | TEX11 | testis expressed 11 [Source:HGNC Symbol;Acc:HGNC:11733] | 8936 | -0.227 | -0.3266 | No |
| 235 | SHH | sonic hedgehog signaling molecule [Source:HGNC Symbol;Acc:HGNC:10848] | 8941 | -0.227 | -0.3249 | No |
| 236 | SIRT1 | sirtuin 1 [Source:HGNC Symbol;Acc:HGNC:14929] | 8947 | -0.227 | -0.3233 | No |
| 237 | SMAD4 | SMAD family member 4 [Source:HGNC Symbol;Acc:HGNC:6770] | 9035 | -0.235 | -0.3282 | No |
| 238 | BSG | basigin (Ok blood group) [Source:HGNC Symbol;Acc:HGNC:1116] | 9043 | -0.235 | -0.3267 | No |
| 239 | PGR | progesterone receptor [Source:HGNC Symbol;Acc:HGNC:8910] | 9048 | -0.235 | -0.3249 | No |
| 240 | PHLDA2 | pleckstrin homology like domain family A member 2 [Source:HGNC Symbol;Acc:HGNC:12385] | 9059 | -0.237 | -0.3237 | No |
| 241 | HAND1 | heart and neural crest derivatives expressed 1 [Source:HGNC Symbol;Acc:HGNC:4807] | 9082 | -0.238 | -0.3233 | No |
| 242 | UBB | ubiquitin B [Source:HGNC Symbol;Acc:HGNC:12463] | 9142 | -0.243 | -0.3259 | No |
| 243 | FKBP4 | FKBP prolyl isomerase 4 [Source:HGNC Symbol;Acc:HGNC:3720] | 9205 | -0.248 | -0.3286 | No |
| 244 | BAK1 | BCL2 antagonist/killer 1 [Source:HGNC Symbol;Acc:HGNC:949] | 9266 | -0.253 | -0.3312 | No |
| 245 | NCOA1 | nuclear receptor coactivator 1 [Source:HGNC Symbol;Acc:HGNC:7668] | 9270 | -0.253 | -0.3292 | No |
| 246 | NUP107 | nucleoporin 107 [Source:HGNC Symbol;Acc:HGNC:29914] | 9274 | -0.253 | -0.3272 | No |
| 247 | MME | membrane metalloendopeptidase [Source:HGNC Symbol;Acc:HGNC:7154] | 9287 | -0.255 | -0.3259 | No |
| 248 | FANCA | FA complementation group A [Source:HGNC Symbol;Acc:HGNC:3582] | 9293 | -0.255 | -0.3241 | No |
| 249 | BAX | "BCL2 associated X, apoptosis regulator [Source:HGNC Symbol;Acc:HGNC:959]" | 9323 | -0.257 | -0.3241 | No |
| 250 | GJA1 | gap junction protein alpha 1 [Source:HGNC Symbol;Acc:HGNC:4274] | 9325 | -0.258 | -0.3219 | No |
| 251 | ARNT | aryl hydrocarbon receptor nuclear translocator [Source:HGNC Symbol;Acc:HGNC:700] | 9427 | -0.267 | -0.3276 | No |
| 252 | ITGB8 | integrin subunit beta 8 [Source:HGNC Symbol;Acc:HGNC:6163] | 9442 | -0.268 | -0.3264 | No |
| 253 | CBL | Cbl proto-oncogene [Source:HGNC Symbol;Acc:HGNC:1541] | 9479 | -0.272 | -0.3269 | No |
| 254 | H3-3A | H3.3 histone A [Source:HGNC Symbol;Acc:HGNC:4764] | 9503 | -0.274 | -0.3263 | No |
| 255 | TSPY2 | testis specific protein Y-linked 2 [Source:HGNC Symbol;Acc:HGNC:23924] | 9518 | -0.276 | -0.3250 | No |
| 256 | BMP7 | bone morphogenetic protein 7 [Source:HGNC Symbol;Acc:HGNC:1074] | 9544 | -0.278 | -0.3245 | No |
| 257 | CYP27B1 | cytochrome P450 family 27 subfamily B member 1 [Source:HGNC Symbol;Acc:HGNC:2606] | 9582 | -0.282 | -0.3250 | No |
| 258 | NCOA4 | nuclear receptor coactivator 4 [Source:HGNC Symbol;Acc:HGNC:7671] | 9589 | -0.282 | -0.3230 | No |
| 259 | HTRA1 | HtrA serine peptidase 1 [Source:HGNC Symbol;Acc:HGNC:9476] | 9692 | -0.291 | -0.3286 | No |
| 260 | LGR4 | leucine rich repeat containing G protein-coupled receptor 4 [Source:HGNC Symbol;Acc:HGNC:13299] | 9719 | -0.294 | -0.3281 | No |
| 261 | ACE | angiotensin I converting enzyme [Source:HGNC Symbol;Acc:HGNC:2707] | 9720 | -0.294 | -0.3255 | No |
| 262 | RHOBTB3 | Rho related BTB domain containing 3 [Source:HGNC Symbol;Acc:HGNC:18757] | 9783 | -0.300 | -0.3278 | No |
| 263 | NEUROG1 | neurogenin 1 [Source:HGNC Symbol;Acc:HGNC:7764] | 9856 | -0.307 | -0.3308 | No |
| 264 | MDFI | MyoD family inhibitor [Source:HGNC Symbol;Acc:HGNC:6967] | 9923 | -0.314 | -0.3333 | Yes |
| 265 | PTX3 | pentraxin 3 [Source:HGNC Symbol;Acc:HGNC:9692] | 9943 | -0.316 | -0.3320 | Yes |
| 266 | NOS3 | nitric oxide synthase 3 [Source:HGNC Symbol;Acc:HGNC:7876] | 9980 | -0.319 | -0.3321 | Yes |
| 267 | BMP6 | bone morphogenetic protein 6 [Source:HGNC Symbol;Acc:HGNC:1073] | 10021 | -0.323 | -0.3325 | Yes |
| 268 | ADAM20 | ADAM metallopeptidase domain 20 [Source:HGNC Symbol;Acc:HGNC:199] | 10028 | -0.324 | -0.3301 | Yes |
| 269 | HSP90AB1 | heat shock protein 90 alpha family class B member 1 [Source:HGNC Symbol;Acc:HGNC:5258] | 10060 | -0.327 | -0.3297 | Yes |
| 270 | CDX2 | caudal type homeobox 2 [Source:HGNC Symbol;Acc:HGNC:1806] | 10078 | -0.328 | -0.3281 | Yes |
| 271 | DNAAF11 | dynein axonemal assembly factor 11 [Source:HGNC Symbol;Acc:HGNC:16725] | 10091 | -0.330 | -0.3262 | Yes |
| 272 | NR2F2 | nuclear receptor subfamily 2 group F member 2 [Source:HGNC Symbol;Acc:HGNC:7976] | 10129 | -0.334 | -0.3262 | Yes |
| 273 | RARA | retinoic acid receptor alpha [Source:HGNC Symbol;Acc:HGNC:9864] | 10141 | -0.335 | -0.3241 | Yes |
| 274 | SRD5A2 | steroid 5 alpha-reductase 2 [Source:HGNC Symbol;Acc:HGNC:11285] | 10149 | -0.336 | -0.3217 | Yes |
| 275 | MAP2K1 | mitogen-activated protein kinase kinase 1 [Source:HGNC Symbol;Acc:HGNC:6840] | 10197 | -0.340 | -0.3225 | Yes |
| 276 | GCM1 | glial cells missing transcription factor 1 [Source:HGNC Symbol;Acc:HGNC:4197] | 10236 | -0.345 | -0.3225 | Yes |
| 277 | NR5A1 | nuclear receptor subfamily 5 group A member 1 [Source:HGNC Symbol;Acc:HGNC:7983] | 10308 | -0.351 | -0.3250 | Yes |
| 278 | CTSB | cathepsin B [Source:HGNC Symbol;Acc:HGNC:2527] | 10313 | -0.352 | -0.3222 | Yes |
| 279 | SYCP2 | synaptonemal complex protein 2 [Source:HGNC Symbol;Acc:HGNC:11490] | 10370 | -0.358 | -0.3236 | Yes |
| 280 | PRDM1 | PR/SET domain 1 [Source:HGNC Symbol;Acc:HGNC:9346] | 10374 | -0.358 | -0.3206 | Yes |
| 281 | PLAC1 | placenta enriched 1 [Source:HGNC Symbol;Acc:HGNC:9044] | 10428 | -0.364 | -0.3217 | Yes |
| 282 | CASP8 | caspase 8 [Source:HGNC Symbol;Acc:HGNC:1509] | 10442 | -0.366 | -0.3195 | Yes |
| 283 | PRKACG | protein kinase cAMP-activated catalytic subunit gamma [Source:HGNC Symbol;Acc:HGNC:9382] | 10466 | -0.370 | -0.3180 | Yes |
| 284 | RBM15 | RNA binding motif protein 15 [Source:HGNC Symbol;Acc:HGNC:14959] | 10499 | -0.373 | -0.3173 | Yes |
| 285 | CASP2 | caspase 2 [Source:HGNC Symbol;Acc:HGNC:1503] | 10506 | -0.374 | -0.3145 | Yes |
| 286 | RETN | resistin [Source:HGNC Symbol;Acc:HGNC:20389] | 10511 | -0.375 | -0.3115 | Yes |
| 287 | CCNF | cyclin F [Source:HGNC Symbol;Acc:HGNC:1591] | 10548 | -0.378 | -0.3111 | Yes |
| 288 | CSDE1 | cold shock domain containing E1 [Source:HGNC Symbol;Acc:HGNC:29905] | 10559 | -0.379 | -0.3085 | Yes |
| 289 | AMHR2 | anti-Mullerian hormone receptor type 2 [Source:HGNC Symbol;Acc:HGNC:465] | 10571 | -0.381 | -0.3060 | Yes |
| 290 | MMP19 | matrix metallopeptidase 19 [Source:HGNC Symbol;Acc:HGNC:7165] | 10652 | -0.388 | -0.3090 | Yes |
| 291 | STK3 | serine/threonine kinase 3 [Source:HGNC Symbol;Acc:HGNC:11406] | 10656 | -0.388 | -0.3058 | Yes |
| 292 | DNAJB6 | DnaJ heat shock protein family (Hsp40) member B6 [Source:HGNC Symbol;Acc:HGNC:14888] | 10702 | -0.394 | -0.3059 | Yes |
| 293 | PTPRN | protein tyrosine phosphatase receptor type N [Source:HGNC Symbol;Acc:HGNC:9676] | 10712 | -0.395 | -0.3032 | Yes |
| 294 | NOTCH2 | notch receptor 2 [Source:HGNC Symbol;Acc:HGNC:7882] | 10720 | -0.395 | -0.3002 | Yes |
| 295 | EREG | epiregulin [Source:HGNC Symbol;Acc:HGNC:3443] | 10778 | -0.403 | -0.3012 | Yes |
| 296 | HSPA5 | heat shock protein family A (Hsp70) member 5 [Source:HGNC Symbol;Acc:HGNC:5238] | 10820 | -0.407 | -0.3009 | Yes |
| 297 | ANXA1 | annexin A1 [Source:HGNC Symbol;Acc:HGNC:533] | 10824 | -0.407 | -0.2976 | Yes |
| 298 | ADAM19 | ADAM metallopeptidase domain 19 [Source:HGNC Symbol;Acc:HGNC:197] | 10838 | -0.409 | -0.2950 | Yes |
| 299 | REN | renin [Source:HGNC Symbol;Acc:HGNC:9958] | 10889 | -0.416 | -0.2953 | Yes |
| 300 | PLEKHA1 | pleckstrin homology domain containing A1 [Source:HGNC Symbol;Acc:HGNC:14335] | 10899 | -0.416 | -0.2924 | Yes |
| 301 | PLG | plasminogen [Source:HGNC Symbol;Acc:HGNC:9071] | 10939 | -0.423 | -0.2918 | Yes |
| 302 | GATA1 | GATA binding protein 1 [Source:HGNC Symbol;Acc:HGNC:4170] | 10958 | -0.425 | -0.2894 | Yes |
| 303 | RAB13 | "RAB13, member RAS oncogene family [Source:HGNC Symbol;Acc:HGNC:9762]" | 10961 | -0.425 | -0.2859 | Yes |
| 304 | FOXF2 | forkhead box F2 [Source:HGNC Symbol;Acc:HGNC:3810] | 10975 | -0.427 | -0.2831 | Yes |
| 305 | REC8 | REC8 meiotic recombination protein [Source:HGNC Symbol;Acc:HGNC:16879] | 11016 | -0.432 | -0.2825 | Yes |
| 306 | GATA2 | GATA binding protein 2 [Source:HGNC Symbol;Acc:HGNC:4171] | 11058 | -0.439 | -0.2819 | Yes |
| 307 | STK4 | serine/threonine kinase 4 [Source:HGNC Symbol;Acc:HGNC:11408] | 11074 | -0.441 | -0.2792 | Yes |
| 308 | MED1 | mediator complex subunit 1 [Source:HGNC Symbol;Acc:HGNC:9234] | 11146 | -0.450 | -0.2809 | Yes |
| 309 | HSD17B2 | hydroxysteroid 17-beta dehydrogenase 2 [Source:HGNC Symbol;Acc:HGNC:5211] | 11148 | -0.451 | -0.2770 | Yes |
| 310 | BMPR2 | bone morphogenetic protein receptor type 2 [Source:HGNC Symbol;Acc:HGNC:1078] | 11174 | -0.454 | -0.2750 | Yes |
| 311 | UTF1 | undifferentiated embryonic cell transcription factor 1 [Source:HGNC Symbol;Acc:HGNC:12634] | 11200 | -0.457 | -0.2730 | Yes |
| 312 | FOSL1 | "FOS like 1, AP-1 transcription factor subunit [Source:HGNC Symbol;Acc:HGNC:13718]" | 11222 | -0.460 | -0.2706 | Yes |
| 313 | INHBA | inhibin subunit beta A [Source:HGNC Symbol;Acc:HGNC:6066] | 11237 | -0.462 | -0.2677 | Yes |
| 314 | PTPN11 | protein tyrosine phosphatase non-receptor type 11 [Source:HGNC Symbol;Acc:HGNC:9644] | 11260 | -0.465 | -0.2653 | Yes |
| 315 | PPARD | peroxisome proliferator activated receptor delta [Source:HGNC Symbol;Acc:HGNC:9235] | 11301 | -0.471 | -0.2644 | Yes |
| 316 | FOXO3 | forkhead box O3 [Source:HGNC Symbol;Acc:HGNC:3821] | 11305 | -0.472 | -0.2604 | Yes |
| 317 | TMF1 | TATA element modulatory factor 1 [Source:HGNC Symbol;Acc:HGNC:11870] | 11328 | -0.475 | -0.2580 | Yes |
| 318 | NKX3-1 | NK3 homeobox 1 [Source:HGNC Symbol;Acc:HGNC:7838] | 11348 | -0.478 | -0.2553 | Yes |
| 319 | ALOX15B | arachidonate 15-lipoxygenase type B [Source:HGNC Symbol;Acc:HGNC:434] | 11394 | -0.486 | -0.2546 | Yes |
| 320 | STK11 | serine/threonine kinase 11 [Source:HGNC Symbol;Acc:HGNC:11389] | 11451 | -0.496 | -0.2547 | Yes |
| 321 | KDR | kinase insert domain receptor [Source:HGNC Symbol;Acc:HGNC:6307] | 11454 | -0.496 | -0.2505 | Yes |
| 322 | COL9A3 | collagen type IX alpha 3 chain [Source:HGNC Symbol;Acc:HGNC:2219] | 11477 | -0.499 | -0.2479 | Yes |
| 323 | KITLG | KIT ligand [Source:HGNC Symbol;Acc:HGNC:6343] | 11516 | -0.503 | -0.2465 | Yes |
| 324 | ADM | adrenomedullin [Source:HGNC Symbol;Acc:HGNC:259] | 11543 | -0.506 | -0.2441 | Yes |
| 325 | RPS6 | ribosomal protein S6 [Source:HGNC Symbol;Acc:HGNC:10429] | 11662 | -0.526 | -0.2488 | Yes |
| 326 | SF1 | splicing factor 1 [Source:HGNC Symbol;Acc:HGNC:12950] | 11664 | -0.527 | -0.2443 | Yes |
| 327 | NSDHL | NAD(P) dependent steroid dehydrogenase-like [Source:HGNC Symbol;Acc:HGNC:13398] | 11670 | -0.527 | -0.2400 | Yes |
| 328 | STC2 | stanniocalcin 2 [Source:HGNC Symbol;Acc:HGNC:11374] | 11746 | -0.540 | -0.2413 | Yes |
| 329 | EPAS1 | endothelial PAS domain protein 1 [Source:HGNC Symbol;Acc:HGNC:3374] | 11758 | -0.542 | -0.2374 | Yes |
| 330 | HOXB13 | homeobox B13 [Source:HGNC Symbol;Acc:HGNC:5112] | 11766 | -0.544 | -0.2331 | Yes |
| 331 | HIF1A | hypoxia inducible factor 1 subunit alpha [Source:HGNC Symbol;Acc:HGNC:4910] | 11833 | -0.556 | -0.2335 | Yes |
| 332 | PTEN | phosphatase and tensin homolog [Source:HGNC Symbol;Acc:HGNC:9588] | 11836 | -0.556 | -0.2288 | Yes |
| 333 | BCL2L11 | BCL2 like 11 [Source:HGNC Symbol;Acc:HGNC:994] | 11867 | -0.564 | -0.2262 | Yes |
| 334 | BPTF | bromodomain PHD finger transcription factor [Source:HGNC Symbol;Acc:HGNC:3581] | 11870 | -0.565 | -0.2214 | Yes |
| 335 | HS6ST1 | heparan sulfate 6-O-sulfotransferase 1 [Source:HGNC Symbol;Acc:HGNC:5201] | 11932 | -0.579 | -0.2211 | Yes |
| 336 | NASP | nuclear autoantigenic sperm protein [Source:HGNC Symbol;Acc:HGNC:7644] | 11970 | -0.587 | -0.2189 | Yes |
| 337 | ADGRG1 | adhesion G protein-coupled receptor G1 [Source:HGNC Symbol;Acc:HGNC:4512] | 11980 | -0.590 | -0.2144 | Yes |
| 338 | YBX3 | Y-box binding protein 3 [Source:HGNC Symbol;Acc:HGNC:2428] | 12024 | -0.599 | -0.2126 | Yes |
| 339 | LIF | LIF interleukin 6 family cytokine [Source:HGNC Symbol;Acc:HGNC:6596] | 12085 | -0.615 | -0.2119 | Yes |
| 340 | PDGFA | platelet derived growth factor subunit A [Source:HGNC Symbol;Acc:HGNC:8799] | 12104 | -0.621 | -0.2079 | Yes |
| 341 | EGFR | epidermal growth factor receptor [Source:HGNC Symbol;Acc:HGNC:3236] | 12109 | -0.622 | -0.2027 | Yes |
| 342 | ARID5B | AT-rich interaction domain 5B [Source:HGNC Symbol;Acc:HGNC:17362] | 12187 | -0.640 | -0.2032 | Yes |
| 343 | HSD17B3 | hydroxysteroid 17-beta dehydrogenase 3 [Source:HGNC Symbol;Acc:HGNC:5212] | 12253 | -0.662 | -0.2026 | Yes |
| 344 | GATA3 | GATA binding protein 3 [Source:HGNC Symbol;Acc:HGNC:4172] | 12276 | -0.670 | -0.1984 | Yes |
| 345 | MMP14 | matrix metallopeptidase 14 [Source:HGNC Symbol;Acc:HGNC:7160] | 12286 | -0.672 | -0.1932 | Yes |
| 346 | WNT4 | Wnt family member 4 [Source:HGNC Symbol;Acc:HGNC:12783] | 12331 | -0.686 | -0.1907 | Yes |
| 347 | GLI1 | GLI family zinc finger 1 [Source:HGNC Symbol;Acc:HGNC:4317] | 12333 | -0.687 | -0.1847 | Yes |
| 348 | SAFB2 | scaffold attachment factor B2 [Source:HGNC Symbol;Acc:HGNC:21605] | 12363 | -0.696 | -0.1809 | Yes |
| 349 | SNAI1 | snail family transcriptional repressor 1 [Source:HGNC Symbol;Acc:HGNC:11128] | 12365 | -0.696 | -0.1748 | Yes |
| 350 | NIPBL | NIPBL cohesin loading factor [Source:HGNC Symbol;Acc:HGNC:28862] | 12385 | -0.703 | -0.1701 | Yes |
| 351 | PDGFB | platelet derived growth factor subunit B [Source:HGNC Symbol;Acc:HGNC:8800] | 12389 | -0.704 | -0.1642 | Yes |
| 352 | ASH1L | ASH1 like histone lysine methyltransferase [Source:HGNC Symbol;Acc:HGNC:19088] | 12405 | -0.708 | -0.1591 | Yes |
| 353 | SOCS3 | suppressor of cytokine signaling 3 [Source:HGNC Symbol;Acc:HGNC:19391] | 12453 | -0.725 | -0.1565 | Yes |
| 354 | JUNB | "JunB proto-oncogene, AP-1 transcription factor subunit [Source:HGNC Symbol;Acc:HGNC:6205]" | 12468 | -0.734 | -0.1511 | Yes |
| 355 | CITED1 | Cbp/p300 interacting transactivator with Glu/Asp rich carboxy-terminal domain 1 [Source:HGNC Symbol;Acc:HGNC:1986] | 12493 | -0.746 | -0.1465 | Yes |
| 356 | ADAMTS1 | ADAM metallopeptidase with thrombospondin type 1 motif 1 [Source:HGNC Symbol;Acc:HGNC:217] | 12553 | -0.775 | -0.1444 | Yes |
| 357 | NOTCH1 | notch receptor 1 [Source:HGNC Symbol;Acc:HGNC:7881] | 12555 | -0.776 | -0.1376 | Yes |
| 358 | PSAP | prosaposin [Source:HGNC Symbol;Acc:HGNC:9498] | 12569 | -0.780 | -0.1318 | Yes |
| 359 | CHD7 | chromodomain helicase DNA binding protein 7 [Source:HGNC Symbol;Acc:HGNC:20626] | 12591 | -0.791 | -0.1265 | Yes |
| 360 | STC1 | stanniocalcin 1 [Source:HGNC Symbol;Acc:HGNC:11373] | 12622 | -0.809 | -0.1217 | Yes |
| 361 | VEGFA | vascular endothelial growth factor A [Source:HGNC Symbol;Acc:HGNC:12680] | 12629 | -0.813 | -0.1150 | Yes |
| 362 | PTGS2 | prostaglandin-endoperoxide synthase 2 [Source:HGNC Symbol;Acc:HGNC:9605] | 12641 | -0.820 | -0.1087 | Yes |
| 363 | HES1 | hes family bHLH transcription factor 1 [Source:HGNC Symbol;Acc:HGNC:5192] | 12676 | -0.849 | -0.1039 | Yes |
| 364 | INHBB | inhibin subunit beta B [Source:HGNC Symbol;Acc:HGNC:6067] | 12680 | -0.851 | -0.0967 | Yes |
| 365 | DAZAP1 | DAZ associated protein 1 [Source:HGNC Symbol;Acc:HGNC:2683] | 12686 | -0.854 | -0.0895 | Yes |
| 366 | TIPARP | TCDD inducible poly(ADP-ribose) polymerase [Source:HGNC Symbol;Acc:HGNC:23696] | 12789 | -0.950 | -0.0893 | Yes |
| 367 | MYC | "MYC proto-oncogene, bHLH transcription factor [Source:HGNC Symbol;Acc:HGNC:7553]" | 12801 | -0.970 | -0.0816 | Yes |
| 368 | CCN1 | cellular communication network factor 1 [Source:HGNC Symbol;Acc:HGNC:2654] | 12802 | -0.970 | -0.0731 | Yes |
| 369 | CEBPB | CCAAT enhancer binding protein beta [Source:HGNC Symbol;Acc:HGNC:1834] | 12804 | -0.973 | -0.0646 | Yes |
| 370 | LHB | luteinizing hormone subunit beta [Source:HGNC Symbol;Acc:HGNC:6584] | 12805 | -0.975 | -0.0560 | Yes |
| 371 | INSR | insulin receptor [Source:HGNC Symbol;Acc:HGNC:6091] | 12856 | -1.071 | -0.0505 | Yes |
| 372 | CTNNB1 | catenin beta 1 [Source:HGNC Symbol;Acc:HGNC:2514] | 12866 | -1.119 | -0.0414 | Yes |
| 373 | PCYT1B | "phosphate cytidylyltransferase 1B, choline [Source:HGNC Symbol;Acc:HGNC:8755]" | 12877 | -1.151 | -0.0320 | Yes |
| 374 | ZFP36L1 | ZFP36 ring finger protein like 1 [Source:HGNC Symbol;Acc:HGNC:1107] | 12888 | -1.244 | -0.0218 | Yes |
| 375 | GJB1 | gap junction protein beta 1 [Source:HGNC Symbol;Acc:HGNC:4283] | 12889 | -1.257 | -0.0108 | Yes |
| 376 | ADAM15 | ADAM metallopeptidase domain 15 [Source:HGNC Symbol;Acc:HGNC:193] | 12893 | -1.310 | 0.0006 | Yes |
Table: GSEA details [plain text format]

  

Fig 2: GOBP\_REPRODUCTIVE\_SYSTEM\_DEVELOPMENT      
 Blue-Pink O' Gram in the Space of the Analyzed GeneSet

  

Fig 3: GOBP\_REPRODUCTIVE\_SYSTEM\_DEVELOPMENT: Random ES distribution      
 Gene set null distribution of ES for **GOBP\_REPRODUCTIVE\_SYSTEM\_DEVELOPMENT**
